# PaCATB, a secreted catalase protecting *Podospora anserina* against exogenous oxidative stress

**DOI:** 10.18632/aging.100360

**Published:** 2011-08-14

**Authors:** Sandra Zintel, Dominik Bernhardt, Adelina Rogowska-Wrzesinska, Heinz D. Osiewacz

**Affiliations:** ^1^ Institute of Molecular Biosciences and ‘Cluster of Excellence Macromolecular Complexes’, Department of Biosciences, J.W. Goethe-University, Max-von-Laue-Str. 9, D-60438 Frankfurt am Main, Germany; ^2^ Protein Research Group, Department of Biochemistry and Molecular Biology, University of Southern Denmark, Campus 55, DK-5230 Odense M, Denmark

**Keywords:** Podospora anserina, secreted proteins, oxidative stress, aging and catalases

## Abstract

A differential mass spectrometry analysis of secreted proteins from juvenile and senescent Podospora anserina cultures revealed age-related differences in protein profiles. Among other proteins with decreased abundance in the secretome of senescent cultures a catalase, termed PaCATB, was identified. Genetic modulation of the abundance of PaCATB identified differential effects on the phenotype of the corresponding strains. Deletion of PaCatB resulted in decreased resistance, over-expression in increased resistance against hydrogen peroxide. While the lifespan of the genetically modified strains was found to be unaffected under standard growth conditions, increased exogenous hydrogen peroxide stress in the growth medium markedly reduced the lifespan of the PaCatB deletion strain but extended the lifespan of PaCatB over-expressors. Overall our data identify a component of the secretome of P. anserina as a new effective factor to cope with environmental stress, stress that under natural conditions is constantly applied on organisms and influences aging processes.

## INTRODUCTION

The filamentous fungus *Podospora anserina* represents a well-studied model organism for aging [1-4]. *P. anserina* has a small genome [5] that is completely sequenced [6], is tractable to experimentation [7], and is characterized by a short lifespan of a few weeks [8]. During the process of aging, the phenotype changes: the pigmentation of the mycelium increases, the growth rate and fertility decreases, the peripheral hyphae become slender and undulate and finally burst [9]. Aging in *P. anserina* has been demonstrated to be associated with various pathways including mitochondrial DNA (mtDNA) instability [10-13], respiration [14-17], ROS generation and scavenging [14, 15, 18-22], mitochondrial dynamics [23], and apoptosis [24-26]. It thus is clear that aging in *P. anserina*, as in other organisms, is not mono-factorial, but depends on many factors and a network of different, interacting molecular pathways [27]. In aging research the ‘free radical theory of aging' (FRTA), which states that reactive oxygen species (ROS) generated during normal metabolism are responsible for damaging cellular components and for aging of cells, organs and organisms [28, 29], is one of the major theories and has been extensively studied over decades in various biological systems. It is now well demonstrated that different ROS are generated by different cellular processes (e.g., the respiratory chain) and by specific reactions [30-38]. It is also clear today that low ROS levels are important components in signal transduction and essential for developmental processes. However, increased levels of ROS are excessively damaging all kinds of biomolecules leading to degeneration of biological systems. To avoid imbalanced levels of ROS, all known organisms exhibit a wide variety of scavenging systems like superoxide dismutases, glutathione peroxidases and reductases, peroxiredoxins and catalases [39-41].

While the FRTA is basically addressing the impact of ROS generated by normal metabolism within a cell, biological systems are also challenged by ROS originating from the environment. For instance, food taken up as energy supply can be such a source. Moreover, the generation and secretion of ROS by organisms is a well known strategy to attack competitors in the same environmental niche or to weaken potential hosts prior to infection [42, 43]. In such situations it may be of advantage for organisms to secrete ROS scavengers like catalases to protect themselves against oxidative stress from outside. For instance, deletion of the *cat-3* gene in *Neurospora crassa* as well as deletion of *catB* in *Aspergillus nidulans* increases the sensitivity against hydrogen peroxide. Furthermore, an induction of the catalase activity caused by treatment with exogenous hydrogen peroxide, identified these catalases as part of the oxidative stress response [44-46].

The aim of our study was to explore a potential impact of exogenous oxidative stress and it's relation to extracellular ROS defense and to aging. Towards this goal we performed a comparative secretome analysis. In comparison to secreted proteins from juvenile cultures of *P. anserina*, we identified a ROS scavenging protein, PaCATB, to increase in abundance in the secretome of senescent cultures. The deletion and over-expression of the gene coding for this catalase provided evidence for a role in protecting growing *P. anserina* cultures against environmental oxidative stress and as such has an impact on the lifespan of this aging model.

## MATERIALS AND METHODS

### Cloning procedures and generation of *P. anserina* mutants

The generation of *PaCatB* over-expressing strains (*PaCatB*_OEx1-3) was performed by amplifying the open reading frame of *PaCatB* (plus ~ 500bps terminator region) by PCR using the oligonucleotides PaCatBEx1-1 (5‘-GGGGATCCAT GAAAAGGCTG CTAACG-3’) and PaCatBEx1-2 (5‘-CCAAGCTTAA AAGCTCACCG GCCAAC-3’), introducing *Bam* HI and *Hin* dIII restriction sites (underlined). The amplicon was cloned into the pExMthph backbone (*Bam*HI / *Hin*dIII digested) resulting in the plasmid pPaCatBEx1. In this the *PaCatB* reading frame is under control of a strong constitutive metallothionein promoter [47]. The plasmid was used to transform *P. anserina* wild-type spheroplasts. Transformants were selected for hygromycin-resistance and verified by Southern blot analysis. Three different strains containing one additional copy of *PaCatB* were subsequently further analyzed (*PaCatB*_OEx1-3).

Deletion of *PaCatB* in wild-type strain s was performed according to a previously described method [48]. Briefly, small flanking regions of *PaCatB* were amplified using the 5‘-flank specific oligonucleotides PaCatBKO1-1 (5‘-CCGGTACCCC TTTGCCGGGG GGCGTG-3’) and PaCatBKO1-2 (5‘-CCCTGCAGCT GCTGCCGCTG CCGTGC-3’) introducing *Kpn*I and *Pst* I restriction sites and the 3'-flank specific oligonucleotides PaCatBKO1-3 (5‘-GGACTAGTGG AAAAGGGAAT GGGTTC-3’) and PaCatBKO1-4 (5‘-GGGCGGCCGC ACTAATATAT ATACCG-3’) with *Bcu* I and *Not* I restriction sites. The fragments were cloned into the plasmid pKO4 [48, 49] next to the bifunctional resistance cassette consisting of a blasticidin resistance gene for selection in *Escherichia coli* and a phleomycin resistance cassette for selection in *P. anserina*. The resistance cassette with the flanking regions was excised by degistion with *Not*I and *Kpn*I and transformed into the *E. coli* KS272 strain which contains plasmid pKOBEG [50] and the *PaCatB* gene containing cosmid 19B11 [51]. Homologous recombination between the flanks of the resistance cassette and cosmid 19B11 leads to generation of cosmid Δ19B11, containing the phleomycin-blasticidin cassette with large flanking genomic regions. The cosmid Δ19B11 was used to transform *P. anserina* wild-type spheroplasts. Transformants were selected by growth on phleomycin-containing medium. Successful deletion of *PaCatB* was indicated by phleomycin resistance and hygromycin sensitivity. The correct replacement of the *PaCatB* gene was verified by Southern blot analysis. The selected strain, lacking the *PaCatB* gene and harbouring the phleomycin gene instead, was termed Δ*PaCatB*.

### Transformation of *P. anserina.*

Production, regeneration, and integrative transformation of *P. anserina* spheroplasts was performed as described [52, 53].

### *P. anserina* strains and strain cultivation

In this study the *P. anserina* wild-type strain s [9], the newly generated *PaCatB* over-expressing strains (*PaCatB*_OEx1-3) and the deletion strain (Δ*PaCatB)* were used. All transgenic strains are in the genetic background of wild-type strain s. Strains were grown on standard cornmeal agar (BMM) at 27 °C under constant light [7]. For germination of spores BMM with 60 mM ammonium acetate was used and incubated at 27 °C in the dark for 3 days. All used strains were originating from mono-nucleate ascospores isolated from irregular asci. In order to obtain cultures of a defined age, mycelia pieces from cultures of freshly germinated ascospores were placed on one side of a Petri dish containing PASM (*P. anserina* synthetic medium) [18].

### Growth rates under oxidative stress conditions

For incubation of strains with hydrogen peroxide, mycelia of monokaryotic isolates (wild-type, Δ*PaCatB* and *PaCatB*_OEx1-3: n=8) were grown on *P. anserina* synthetic medium (PASM) [26] containing different hydrogen peroxide concentrations (0-45 mM hydrogen peroxide) for 4 days. Plates were kept in the dark to protect hydrogen peroxide from degradation. Growth rates were assigned daily and calculated as growth rate per day.

### Lifespan determination

Lifespan determination was performed with cultures originating mono-nucleate ascospores (wild-type: n= 66, Δ*PaCatB*: n=20, *PaCatB*_OEx1: n=29, *PaCatB*_OEx2: n=11, *PaCatB*_OEx3: n=14). Mycelial pieces from cultures of freshly germinated ascospores were placed on race tubes containing PASM [19]. Survival curves were calculated allowing the determination of the median lifespan.

### Lifespan determination under oxidative stress

Lifespan determination under oxidative stress was performed as described above. The different isolates were placed on Petri dishes with PASM containing 0.75 mM hydrogen peroxide (wild-type: n=23, Δ*PaCatB*: n=27) or 3 mM hydrogen peroxide (wild-type and *PaCatB*_OEx1-3: n=40).

### Isolation of secreted proteins

Mycelia from wild-type strain s, *PaCatB*_OEx1-3 or Δ*PaCatB* were allowed to overgrow the surface of cellophane foil occupied BMM plates. After an incubation of 3 days at 27 °C under constant light, the cultured mycelia were transferred into tubes of liquid media (150 ml CM) and were incubated for 4 days at 27 °C under constant light without shaking to prevent cell damages and consequential resultant contamination with total cell extract. The filtered liquid medium was enriched by filter tubes (Amicon Ultra-15, Ultracel-3k, Millipore, Schwalbach, Germany). Supernatants were mixed with 1:100 PIC (Protease Inhibitor Cocktail). The secreted proteins were precipitated by adding 2 volumes of ethanol and 2 volumes of acetone for 2 days at -20 °C. After centrifugation (30 min, 4 °C, 15000 rpm FA-45-24-11 fixed angle rotor) 1 ml desalinisation solution (2:1:1 water: ethanol: acetone) was added to the pellet. After 1 min of vortexing, the secreted proteins were incubated over night at -20 °C. This desalinisation procedure was repeated twice. Pellets were dried and resolved with 50 μl protein extraction puffer at room temperature.

### Isolation of whole cell protein

For extraction of whole cell protein, mycelia from different *P. anserina* strains was allowed to overgrow a cellophane foil covered PASM surface for 4 days in the dark. Subsequently, harvested mycelia were pulverized in liquid nitrogen. The protein was isolated from the powder as described [20].

### Two-dimensional gel electrophoresis and silver staining

Secreted protein probes were purified (ReadyPrep™ 2-D Cleanup Kit: Bio-Rad, Munich, Germany) and eluted in IEF buffer (7 M urea, 2 M thiourea, 0.4% dithiothreitol, 2% CHAPS and 0.5% ampholytes at pH 3-10). For the isoelectric focusing, protein (400 μg) was applied to IEF strips (17 cm, pH 3-10, Bio-Rad Munich, Germany). The electro-focusing program was limited to 50 μA per strip and started with 250V for 15 min. Voltage rose rapidly up at 10,000 V for 3 h. This was maintained till 60,000 Vh were obtained. The strips were equilibrated in Tris base (45 mM at pH 8.8, 6 M urea, 2% SDS, 30% glycerol and 2% dithiothreitol) for 10 min and then equilibrated for 10 min in the same buffer with 2.5% iodoacetamide instead of dithiothreitol. The strips were fixed on top of SDS separating polyacrylamide gels (10%) with a 0.5% agarose solution in SDS separation buffer with 0.00067% bromphenol blue. The gels were run at 6 mA/ gel for 3 h followed by 18 mA/ gel for 4 h. The current was set to 20 mA/ gel until the bromphenol blue flew out. The gels were silver stained as previously described [54].

### Western blot analysis

10 μg secreted protein were fractionated by two-phase SDS-PAGE (6-18 % separating gels) according to standard protocol [25]. After electrophoresis, proteins were transferred to PVDF membranes (Immobilon Transfer Membranes, Millipore, Schwalbach, Germany). Blocking, antibody incubation and washing steps were performed according to the Odyssey Western blot analysis handbook (Li-Cor, Lincoln, NE, USA). As primary antibodies the following antibodies were used: Anti-PaCATB (1:15000 dilution) raised against a PaCATB specific synthetic peptide ([Ac]-CRYLGRFPVDEGAE-[OH] (New England Peptide, Gardner, USA). In all analyses, secondary antibodies conjugated with IRDye CW 800 (1:15000 dilution, Li-Cor, Lincoln, NE, USA) were used. The Odyssey infrared scanner (Li-Cor, Lincoln, NE, USA) was used for detection.

### Qualitative ‘in-gel’ catalase activity assay

The ‘in-gel’ catalase stain was performed as described [55] with native gradient PAGE. Following the electrophoresis the gel was washed 3 times 15 min in water. After washing the gel it was incubated 10 min in a 0.003 % H_2_O_2_ solution. Subsequently, the gel was transferred into 30 ml freshly prepared solution containing 2 % potassium ferricyanide and 2 % ferric chloride. The gel tray was gently agitated over a light box until appearance of a green color in the gel. After rinsing the gel with water it was scanned for further analysis.

### Quantitative photometric measurement of catalase activity

The photometric quantification of H_2_O_2_ degradation was performed in quartz glass cuvette (104-QS 0.500, Hellma, Müllheim, Germany) by measuring the absorption at 240 nm in intervals of 10 sec for a total of 10 min. The cuvette was loaded with 300 μl of a 0.01 M potassium phosphate buffer (pH 7.0) and 100μl of a 300 mM H_2_O_2_ solution dissolved in 0.01 M potassium phosphate buffer (pH 7.0) was added. After reaching a constant absorption, 100 μl of a 0.02 μg/μl protein solution, dissolved in 0.01 M potassium phosphate buffer (pH 7.0), was added. The percentage absorption decrease at 240 nm per time, representing the catalase activity, was calculated based on the amount of the linear slope after addition of the protein solution. All samples were measured as triplets in three independent experiments.

### Identification of proteins separated by two-dimensional gel electrophoresis

Protein spots of interest were manually excised from gels and digested with trypsin (Promega Inc., Madison, WI, USA). The resulting peptide mixture was desalted and analyzed using a 4800 Plus MALDI TOF / TOF Analyzer (Applied Biosystems, Foster City, CA, USA) as described before [56]. From the raw data output, peak lists were generated by Data Explorer (Applied Biosystems, Foster City, CA, USA). MS and MS/MS peak lists were combined into search files and used to search protein databases using the Mascot search engine (Matrix Science Ltd, London, UK). Search parameters were as follows: Database: NCBInr 20110602 or UniProt Version 49; Taxonomy: all entries or fungi; Enzyme: trypsin; Allow up to 1 missed cleavage; Fixed modifications: none; Variable modifications: methionine oxidation; Peptide mass tolerance: 70 ppm; and Fragment mass tolerance: 500 ppm. Positively identified proteins have been assigned a significant Mascot score based on the probability that the observed match is a random event and protein scores greater than 56 are significant (P < 0.05).

### Quantitative proteomics using iTRAQ labeling

Identification and quantitation of the secretome of juvenile and senescent *P. anserina* cultures have been achieved by iTRAQ labelling and nanoLC-MS/MS in combination with sample pre-fractionation by SCX (strong cathion exchange) following previously described protocols [57]. Briefly, proteins precipitated from growth medium have been reduced and alkylated and subsequently digested with trypsin endopeptidase. 100 μg of peptides derived from each sample were labeled with iTRAQ 114 and 117 according to manufacturer's instructions and mixed at 1:1 ratio. The mixed sample was separated by SCX into 6 fractions corresponding to 6 elution buffers with different ion force (25, 50, 75, 100, 200 and 500 mM KCl in 30% acetonitrile, pH 3). The iTRAQ labeled peptides from SCX fractions were analyzed using EASY nanoLC system (Proxeon, Denmark) equipped with in-house packed fused silica C18 analytical column (Reprosil, Dr. Maisch, 15 cm, 100 μm, I.D., 375 μm, O.D.). The gradient induced a linear increase of 0-32% acetonitrile in 0.1% formic acid over 50 minutes, at a flow rate of 250 nl/minute. Eluted peptides were sprayed into a LTQ-Orbitrap XL (Thermo Electron, Bremen, Germany) via a nanoelectrospray ion source (Proxeon Biosystems, Odense, Denmark). The mass spectrometer was operated in data-dependent mode automatically switching between MS, MS/MS CID (collision-induced dissociation) and MS/MS HCD (high-energy collision dissociation) modes using a 50000 threshold for ion selection.

The data were processed described before with minor changes [57]. The raw data were analyzed using Proteome Discoverer, version 1.0 and an in-house MASCOT server (version 2.1) (Matrix Science Ltd., London, U.K.) for database searching through the Proteome Discoverer program. The data were searched against the Uniprot *Podospora anserina* sequence database (version 55). Trypsin was used as a cleavage enzyme with a maximum of 2 missed cleavages was allowed. Carbamidomethyl (C) was chosen as a fixed modification. As variable modifications, iTRAQ 4plex (K), iTRAQ 4plex (N-term) and Oxidation (M) were chosen. The data were searched with a peptide mass tolerance of 10 ppm and a fragment mass tolerance of 0.8 Da (CID) and 0.1 Da (HCD). Only proteins identified by at least 2 peptides and mascot peptide ion scores of at least 20 were considered. The quantitative results were normalized on protein median.

## RESULTS

### Identification of a secreted catalase which decreases in abundance during aging of *P. anserina*

**Figure 1:**
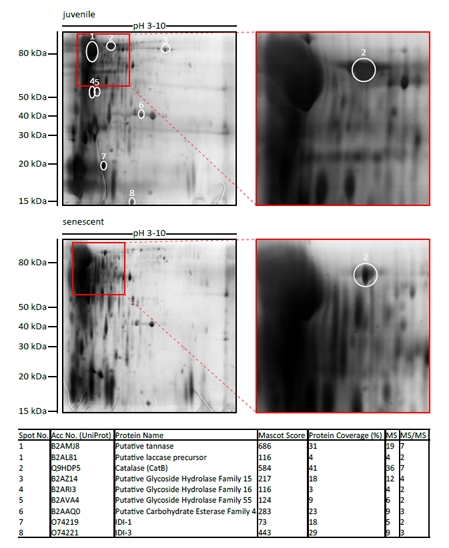
Identification of age-regulated secreted proteins For identification of age-regulated proteins, 400 μg secreted proteins of either juvenile or senescent wild-type were first separated by 2D SDS-PAGE using IPG strips of pH 3-10. The gels were silver stained and spots with differing abundance between juvenile and senescent probes were picked and analyzed by mass spectrometry. The table shows the proteins which were identified in spots 1-8. Different parameters clarifying protein identification by MS are indicated: accession number from UniProt database, mascot score (> 56 is significant at P < 0.05), % sequence coverage, no of matched peptides (MS), and no. of sequenced peptides (MS/MS).

In order to identify age-regulated proteins in the secretome of *P. anserina*, secreted proteins from juvenile and senescent wild-type cultures were isolated and analyzed by 2D SDS-PAGE and mass spectrometry (Figure 1). Along with secreted age-regulated enzymes like hydrolases, one laccase and proteins induced during incompatibility reactions, a catalase, named PaCATB, was identified. To confirm the expression changes observed by 2D SDS-PAGE isobaric tags (iTRAQ) combined with nano liquid chromatography and mass spectrometry (nanoLC-MS/MS) were used. Relative quantitation revealed that PaCATB abundance in secretome of senescent wild-type was 3.4 times decreased in comparison to secretome probe from juvenile cultures (Table 1).

**Table 1:** Relative quantitation of juvenile and senescent secretome probes Whole The 13 proteins found to be differentially secreted between juvenile and senescent cultures of *P. anserina* using iTRAQ and nanoLC-MS/MS. The accession number for each protein is listed along with the number of unique quantified peptides assigned to each protein. The overall protein ratio measured by iTRAQ is indicated for each protein. These values are based on the total peptide information obtained for the individual proteins in all analyzed SCX fractions. Protein score is the sum of the scores of the individual peptides

Acc No. (UniProt)	Protein Name	Protein coverage (%)	No of quantified peptides	Protein Score	Quant. Ratio (juv/sen)
B2AS94	Acyl CoA binding protein	24.27	5	140	10.8
B2ADW0	Putative protein of unknown function	7.23	5	141	9.4
B2AL81	Putative laccase precursor	2.68	20	241	3.4
**Q9HDP5**	**Catalase (PaCatB)**	**9.69**	**6**	**146**	**3.4**
B2ATI7	Putative oxalate decarboxylase	2.31	3	98	3.0
B2APL6	Putative ATP-dependent RNA helicase ATP-dependent RNA helicase RhlE	2.78	19	262	3.0
B2AFG3	Putative protein of unknown function	9.48	3	78	2.9
B2AP41	Putative glycoside hydrolase Family 16	5.09	4	156	2.8
B2B0H8	Putative ATP dependent RNA helicase superfamily II RNA helicase	1.57	6	61	2.7
B2B476	Putative protein of unknown function	4.59	3	75	2.6
B2B5J4	Putative guanyl-specific ribonuclease N1 precursor	7.45	5	91	2.4
B2ADM3	Putative glycoside hydrolase Family 15	2.01	2	22	2.4
B2AB03	Putative tyrosinase	2.33	7	73	0.5

### *The genome of P. anserina* encodes five putative catalases

**Figure 2:**
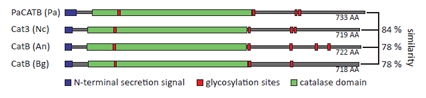
Schematic representation of PaCATB and different homologues The schematic protein sequence of different PaCATB homologues is shown with their N-terminal secretion signal, glycolysation sites and catalase domain. *Podospora anserina* (Pa) PaCATB: Q9HDP5 (UniProt); *Neurospora crassa* (Nc) Cat3: Q9C169 (UniProt); *Aspergillus nidulans* (An) CatB: P78619 (UniProt); *Blumeria graminis* (Bg) CatB: AAl56982 (EMBL database). The amino acid similarity is indicated as percentage of the PaCATB amino acid sequence.

*P. anserina* encodes five putative catalases with different predicted localizations (*http://podospora.igmors.u-psud.fr, http://wolfpsort.org*). Two of them are small subunit catalases within the peroxisome (PaCATP1) or the cytosol (PaCATP2). In addition, a catalase-peroxidase (PaCAT2) is localized in the cytosol. Furthermore, two large putative catalases, PaCATA and PaCATB, are encoded by the *P. anserina* genome. PaCATA is predicted to be bound to the plasma membrane whereas PaCATB contains a putative secretion sequence. Homologues of PaCATB are known in various ascomycetes such as *Neurospora crassa* (Cat-3), *Aspergillus nidulans* (CatB) and *Blumeria graminis* (CatB) with a high similarity (e-value: 0; http://www.ncbi.nlm.nih.gov) (Figure 2). They all contain an N-terminal secretion signal, a highly conserved catalase domain and several glycosylation sites.

### Verification of transgenic *PaCatB P. anserina* strains

In order to investigate the role of the putative secreted and age-regulated catalase PaCATB in lifespan control and ROS scavenging, we set out to generate strains in which *PaCatB* is deleted or over-expressed. One *PaCatB* deletion strain (Δ*PaCatB*) and three independent *PaCatB* over-expression strains (*PaCatB*_OEx1-3) containing one extra copy of *PaCatB* under the control of the strong constitutive metallothionein promoter [47] integrated at different sites in the genome of *P. anserina* were selected and subsequently analyzed.

**Figure 3:**
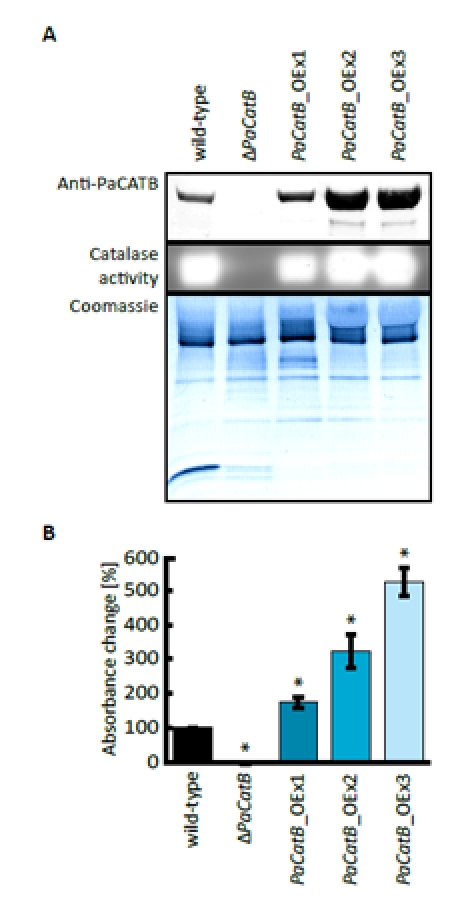
Verification of *PaCatB* deletion and *PaCatB* over-expression strains. A Probes of 10 μg secreted protein of wild-type, *PaCatB* deletion strain (Δ*PaCatB*) and the three independent *PaCatB* over-expression strains (*PaCatB*_OEx1-3) were analyzed by Western blot analysis and an ‘in-gel’ catalase activity assay (6-20 % separating gel). PaCATB was detected via a specific antibody against catalase B in *P. anserina* (Anti-PaCATB). The Coomassie stained PVDF membrane was used as a loading control of secreted protein probes. The activity of PaCATB is visualized as highlighted bands. Accession numbers: PaCATB: Q9HDP5 (UniProt). **B** Catalase activity was quantified by measuring the photometric hydrogen peroxide degradation of secreted protein probes of wild-type (n=2, p-value: p<0.01), *PaCatB* deletion strain (Δ*PaCatB*, n=9) and the three independent *PaCatB* over-expression strains (*PaCatB*_OEx1-3, n=9). The percentage absorption decrease at 240 nm per time represents the catalase activity. Error bars are ± SEM.

First, PaCATB protein abundance and activity of the three *PaCatB* over-expression strains (*PaCatB*_OEx1-3) and the *PaCatB* deletion strain (Δ*PaCatB*) were verified (Figure 3 A-B). PaCATB abundance was investigated by Western blot analysis with secreted protein probes of *PaCatB* deletion strain and over-expression strains using a newly generated specific PaCATB antibody (Figure 3 A). PaCATB levels are increased in *PaCatB* over-expressors while PaCATB protein is undetectable in the *PaCatB* deletion strain (Figure 3 A).Consistently, no PaCATB activity was identified in secreted protein probes of the deletion strain while the *PaCatB* over-expressors showed an increased PaCATB activity in the ‘in-gel’ catalase activity assay (Figure 3 A). In order to verify the activity of PaCATB, we next measured the hydrogen peroxide decomposition in these transgenetic *PaCatB* strains (Figure 3 B). The deletion strain (Δ*PaCatB*) completely failed to decompose hydrogen peroxide while the three over-expressors (*PaCatB*_OEx1-3) decomposed hydrogen peroxide with a 2- to 6-fold higher rate compared to the wild-type (Figure 3 B).

### Impact of PaCATB on resistance against exogenous hydrogen peroxide stress

**Figure 4:**
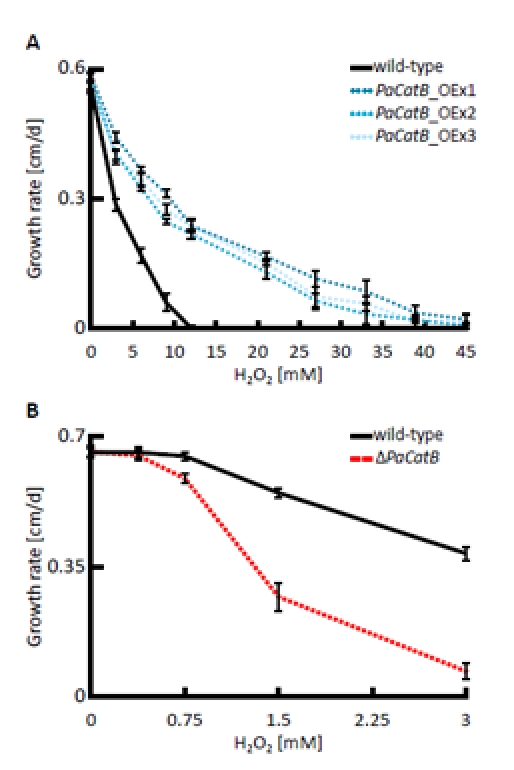
Analysis of hydrogen peroxide tolerance The growth rate of wild-type, *PaCatB* deletion strain (Δ*PaCatB*) and the three independent *PaCatB* over-expression strains (*PaCatB*_OEx1-3) were investigated under oxidative stress. **A** Growth rates of *PaCatB* over-expression strains (n=8) were measured on PASM containing 0, 3, 6, 9, 12, 21, 27, 33, 39 and 45 mM hydrogen peroxide and were statistically significant for 3, 6 and 9 mM (*PaCatB*_OEx1-3: p<0.01), 12, 21, 27 and 33 mM (*PaCatB*_OEx1-3: p<0.01) and 39 mM hydrogen peroxide (*PaCatB*_OEx1-2: p<0.01).**B** Growth rates of *PaCatB* deletion strain (n=8) were measured on PASM containing 0, 0.375, 0.75, 1.5 and 3 mM hydrogen peroxide and were statistically significant for 0.75, 1.5 und 3 mM hydrogen peroxide (p-value: p<0.01). Error bars are ± SEM.

Next we tested the resistance of *PaCatB* deletion strain and *PaCatB* over-expression strains against hydrogen peroxide added to the growth medium in different concentrations (Figure 4). Compared to the wild-type strain, growth rates of the different *PaCatB* mutants did not differ on medium without hydrogen peroxide. However, on medium with additional exogenous hydrogen peroxide the *PaCatB* over-expressors (*PaCatB*_OEx1-3) displayed significant increased growth rates (Figure 4 A). These strains were able to grow on medium with high levels of hydrogen peroxide while the wild-type was not able to grow under such conditions. In contrast, the deletion strain (Δ*PaCatB*) was characterized by an increased sensitivity against low levels of exogenous hydrogen peroxide (Figure 4 B).

### Modulation of PaCATB abundance affects lifespan at increased exogenous hydrogen peroxide stress

**Figure 5:**
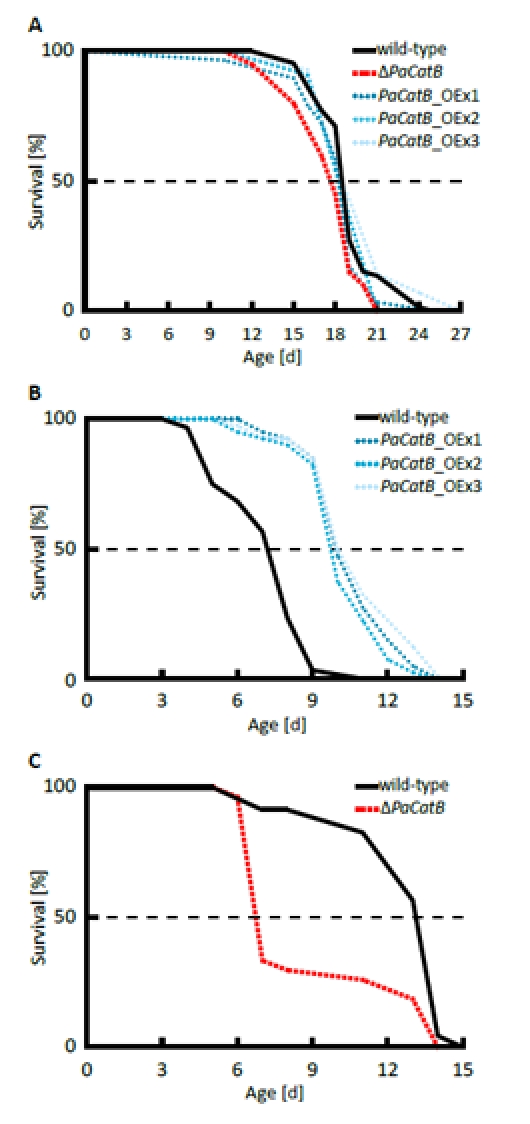
Determination of median lifespan under normal and oxidative stress condition. A Median lifespan under normal standard condition (PASM without hydrogen peroxide) was analyzed for the wild-type (n=66), Δ*PaCatB* (n=20), *PaCatB*_OEx1 (n=29), *PaCatB*_OEx2 (n=11) and *PaCatB*_OEx3 (n=14). Neither median lifespan of Δ*PaCatB* (17.67 d) nor median lifespan of *PaCatB* over-expression strains (*PaCatB*_OEx1:18.23 d, *PaCatB*_OEx2:18.25 d and *PaCatB*_OEx3:18.5 d) were differentially to the median lifespan of the wild-type (18.48 d). P values were determined using the Superior Performing Statistical Software (SPSS) 16 (SPSS Incorporated). **B** Median lifespan under oxidative stress (PASM containing 3 mM hydrogen peroxide) was analyzed from the wild-type (n=40), *PaCatB*_OEx1-3 (n=40). The median lifespan for wild-type was 7.2 d, *PaCatB*_OEx1:10 d, *PaCatB*_OEx2:9.72 d and *PaCatB*_OEx3:9.93 d. Lifespan was 35 % significant increased (p-value: p<0.001). **C** Median lifespan under oxidative stress (PASM containing 0.75 mM hydrogen peroxide) was analyzed from the wild-type (n=23) and Δ*PaCatB* (n=27). The median lifespan for wild-type was 13.12 d and for Δ*PaCatB* 6.7 d. Lifespan was 49 % significant reduced (p-value: p<0.001).

In order to address the impact of the modulation of PaCATB abundance and activity on aging we next determined the lifespan of the different strains under different oxidative growth conditions. On standard medium without added hydrogen peroxide the *PaCatB* deletion and *PaCatB* over-expressing strains and the wild-type are characterized by similar lifespans (Figure 5 A). In contrast, clear differences were observed when strains were grown on media with increased hydrogen peroxide (Figure 5 B and C). On hydrogen peroxide supplemented medium the *PaCatB* over-expression strains showed an increased median lifespan (+35 %) compared to the wild-type (Figure 5 B). In contrast, *PaCatB* deletion resulted in a highly significant reduced median lifespan (-49 %) (Figure 5 C).

### Induction of PaCATB activity as a response to hydrogen peroxide mediated stress

**Figure 6:**
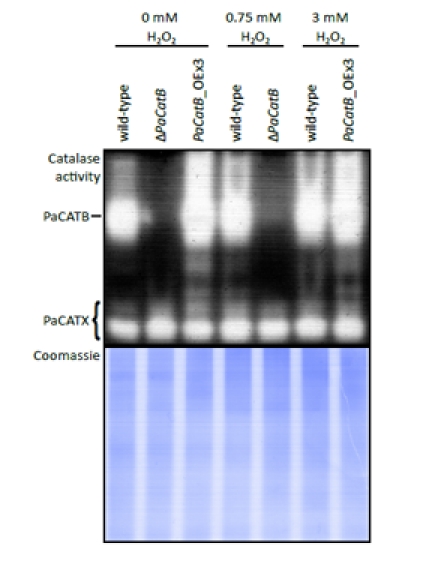
Examination of catalase activity during oxidative stress conditions Whole cell protein was extracted from mycelia grown on PASM for four days in the dark on media containing different hydrogen peroxide concentrations (0, 0.75, 3 mM H_2_O_2_). 40 μg protein of wild-type strain, deletion strain (Δ*PaCatB*) and an over-expressing strain (*PaCatB*_OEx3) were analyzed by an ‘in-gel’ catalase activity assay (10 % separating gel). Catalase activities differing from PaCATB are marked as PaCATX. A Coomassie stained gel loaded with 40 μg of the same protein aliquot was used as a loading control.

To determine the induction of PaCATB activity and thereby an active mechanism of protection against hydrogen peroxide, catalase activity of whole protein extracts from different strains were analyzed (Figure 6).

These strains were grown on media containing different amounts of hydrogen peroxide (0, 0.75 and 3 mM). When grown on media with added hydrogen peroxide, the wild-type strain exhibits an increased catalase B activity compared to the control without oxidative stress. While the wild-type shows an induction of PaCATB activity by hydrogen peroxide stress, the deletion strain proofs that the remaining catalases of *P. anserina* (Figure 6: PaCATX) have no compensatory effects. In the over-expressors no obvious up-regulation of catalase activity can be detected. This is consistent with the transcriptional regulation of the extra *PaCatB* copy which is under the control of a strong constitutive promoter. In these strains, the hydrogen peroxide regulated CATB activity of the endogenous gene most likely is overlaid by the activity of the protein encoded by the transgene.

## DISCUSSION

*P. anserina* is a filamentous fungus that is an extensively studied aging model. Studies with this system were generally taking advantage of the possibility to keep the organism under well defined and controlled conditions in the laboratory and to carry out specifically designed manipulations. In this way the successful identification of a variety of individual components and molecular pathways involved in the control of aging and lifespan was possible (for reviews see: [1-4, 58, 59]). However, although very effective, such studies also bear the risk that certain components escape identification, components and pathways that nevertheless are of importance in the complex network governing biological aging.

In *P. anserina*, the impact of the natural conditions and the interactions with many other microorganisms is only initially investigated [43] but may be of specific relevance for aging. In order to approach this situation, we initiated a study of the secretome of juvenile and senescent *P. anserina* cultures. The rationale behind this strategy was to identify factors secreted and effective in protecting the growing individual against environmental stress.

Comparing the secretome of juvenile and senescent cultures by 2D SDS-PAGE and mass spectrometry identified a number of proteins which change in abundance. Among others, PaCATB abundance decreased in the secretome of senescent cultures. Since catalases are ROS scavenging proteins and oxidative stress is of particular relevance for aging, PaCATB was selected for further studies.

The analyses of strains with altered PaCATB abundance revealed an age-related function of this enzyme. While no effect on lifespan was observed under standard growth conditions when *PaCatB* over-expressors and a deletion strain were compared to the wild-type, there was a clear effect when strains were challenged with increased amounts of hydrogen peroxide in the medium. Strains with increased PaCATB were long-lived and showed an increased tolerance against hydrogen peroxide while those missing PaCATB were short-lived and highly sensitive against this ROS. The sensitivity of the *PaCatB* deletion strain against hydrogen peroxide is consistent with the findings in *A. nidulans* and *N. crassa* catalase deletion strains [44-46].

To the best of our knowledge this is the first report of an effect on tolerance against oxidative stress, aging and lifespan of a secreted isoform of the ROS scavenging system. Such a system may have evolved as a consequence of increased oxidative stress in the natural substrate, herbivorous dung, *P. anserina* is growing on and may be due to the competition for the same natural resources with the many microorganisms living in this ecological niche. The secreted catalase may provide a selective advantage and help to protect against the attack by microorganisms in the immediate environment. Parasitic fungi use ROS as chemical weapons to weaken a potential host prior to successful infection [60, 61]. For *P. anserina* the secreted catalase may indeed provide an efficient protection shield and to allow extraction of enough nutrients from the growth substrate and thus to go successfully through a full reproduction cycle.

In contrast to the role of an ROS scavenging systems outside the cell, there is a huge literature about the impact of cellular ROS scavenging on aging and disease. Since hydrogen peroxide generated inside the cell can cross membranes and age-related increase of intracellular ROS generation also affects extracellular ROS levels a secreted catalase may be important for protection of growing hyphae. In *P. anserina* it is known that in senescent cultures hydrogen peroxide secretion is increased compared to juvenile and middle-aged cultures [23]. Together with a reduction in the abundance of the secreted catalase PaCATB reported in this study, damage of the growing hyphal tips via increased ROS in the environment may in fact be a major reason leading to the observed swelling and burst of these fragile parts of a *P. anserina* culture [62].

Many of the different studies investigating the role of oxidative stress and of ROS scavenging systems are contradictory and the results are taken to question the FRTA [63, 64]. However, it should be emphasized at this point that the various findings that- on first glance- appear to contradict the theory need also be taken with caution because the different biological systems may have developed some special pathways that do not allow full comparability of one system to another one. And more importantly, studies in which the expected outcome is not obtained may be the result of specific compensatory effects that are not considered. For instance, in a recent study with *P. anserina* we generated strains in which a gene coding for the mitochondrial superoxide dismutase (PaSOD3) was over-expressed. Surprisingly, the corresponding strains were not long- but short-lived and showed different kinds of impairments. A more detailed characterization of these mutants could solve part of the counter-intuitive results. *PaSod3* over-expressors were found to be impaired in components of the mitochondrial protein quality control system, like the mitochondrial matrix protease PaCLPP or the heat shock protein PaHSP60, and in mitochondrial peroxiredoxin, a protein converting hydrogen peroxide into water [19]. It appears that ROS generation, signaling, and scavenging are part of a delicate network of interacting pathways with strong impact on aging and development. Reductionistic approaches studying just a single component are therefore prone to raise surprising and counter-intuitive results demonstrating the need for more holistic approaches as they are used in systems biology.
